# Rapid Detection of K1 Hypervirulent *Klebsiella pneumoniae* by MALDI-TOF MS

**DOI:** 10.3389/fmicb.2015.01435

**Published:** 2015-12-21

**Authors:** Yonglu Huang, Jiaping Li, Danxia Gu, Ying Fang, Edward W. Chan, Sheng Chen, Rong Zhang

**Affiliations:** ^1^Department of Clinical Microbiology, Second Affiliated Hospital of Zhejiang UniversityHangzhou, China; ^2^Shenzhen Key Lab for Food Biological Safety Control, Food Safety and Technology Research Center, Hong Kong PolyU Shenzhen Research InstituteShenzhen, China; ^3^State Key Lab of Chirosciences, Department of Applied Biology and Chemical Technology, The Hong Kong Polytechnic UniversityHong Kong, China

**Keywords:** K1 hvKP, MALDI-TOF MS, rapid detection, SVM model, typical spectra

## Abstract

Hypervirulent strains of *Klebsiella pneumoniae* (hvKP) are genetic variants of *K. pneumoniae* which can cause life-threatening community-acquired infection in healthy individuals. Currently, methods for efficient differentiation between classic *K. pneumoniae* (cKP) and hvKP strains are not available, often causing delay in diagnosis and treatment of hvKP infections. To address this issue, we devised a Matrix-assisted laser desorption/ionization time-of-flight (MALDI-TOF) mass spectrometry (MS) approach for rapid identification of K1 hvKP strains. Four standard algorithms, genetic algorithm (GA), support vector machine (SVM), supervised neural network (SNN), and quick classifier (QC), were tested for their power to differentiate between K1 and non-K1 strains, among which SVM was the most reliable algorithm. Analysis of the receiver operating characteristic curves of the interest peaks generated by the SVM model was found to confer highly accurate detection sensitivity and specificity, consistently producing distinguishable profiles for K1 hvKP and non-K1 strains. Of the 43 *K. pneumoniae* modeling strains tested by this approach, all were correctly identified as K1 hvKP and non-K1 capsule type. Of the 20 non-K1 and 17 K1 hvKP validation isolates, the accuracy of K1 hvKP and non-K1 identification was 94.1 and 90.0%, respectively, according to the SVM model. In summary, the MALDI-TOF MS approach can be applied alongside the conventional genotyping techniques to provide rapid and accurate diagnosis, and hence prompt treatment of infections caused by hvKP.

## Introduction

*Klebsiella pneumoniae*, a facultative anaerobic gram-negative bacillus (Fang et al., 2004), is an important opportunistic pathogen associated with both community-acquired and nosocomial infection such as pneumonia, urinary tract infections, septicemia, and wound infections, especially among patients in ICU (Vardakas et al., 2015). The first case of hypervirulent *Klebsiella pneumoniae* (HvKP) infection was reported to have originated from a patient with liver abscess in China in 1980s (Siu et al., 2012). Hvkp is a variant which is morphologically different from the classic strain in terms of appearance of colonies grown on agar plate. HvKP is not only able to cause nosocomial infection in immunocompromised patients, but more importantly, it often causes life-threatening community-acquired (CA) infection in healthy individuals, eliciting a great concern worldwide (Liu et al., 2014). In recent years, the incidence of hvKP infection has increased markedly in various countries including Asia (Zhang et al., 2014), Europe (Decré et al., 2011), and South America (Vila et al., 2011).

Different from classical *K. pneumoniae* (cKP), hvKP has high iron acquisition ability, an increase in capsule production mediated by rmpA/rmpA2, which confers the hypermucoviscous, and association with Mucoviscosity-associated gene A (magA) and are commonly seen in K1, K2, K5, K20, K54, and K57 with K1 and K2 being the most dominant serotypes (Yeh et al., 2010; Shon et al., 2013). Emergence of hvKP strains represents a huge threat to human health (Shen et al., 2013) but methods for efficient differentiation between classic and hvKP strains are not available.

Matrix assisted laser desorption ionization-time of flight mass spectrometry (MALDI-TOF MS) is considered as an efficient tool which can accurately identify both commonly encountered pathogenic bacterial species and microbial pathogens that are difficult to identify, such as yeasts, anaerobes, and fastidious microorganisms (Martiny et al., 2012). Recently, some studies have shown that this technology exhibits the capacity for rapid discrimination of antibiotic resistant strains such as methicillin-resistant *Staphylococcus aureus* (Madhava Charyulu et al., 2012; Hu Yy et al., 2015) and carbapenem-resistant Enterobacteriaceae (Lau et al., 2014) from the sensitive organisms, detection of virulence factors such as *S. aureus* delta-toxin (Gagnaire et al., 2012; Josten et al., 2014), and epidemiological typing (Josten et al., 2013). In this work, we developed a MALDI-TOF MS method for rapid identification of K1 *K. pneumoniae* isolates, and evaluated its reliability in rapid detection of major *K. pneumoniae* virulence factors.

## Materials and methods

### Conventional K1 hvKP identification

A string test was performed to identify hvKP from clinical *K. pneumoniae* strains. A positive string test is defined as the formation of a mucoviscous string of >5 mm in length when using a bacteriology inoculation loop to touch and stretch a colony grown overnight on an blood agar plate at 35°C (Fang et al., 2007). The capsular polysaccharide synthesis virulence genes (K1, K2, K5, K20, K54, and K57) and other relevant genes (*wcaG, rmpA, magA*, and Aerobactin) were amplified through a TPersonal cycler (Biometra, Germany) to further identify K1 hvKP strains. Primer sequence and annealing temperature were shown in Table 1. PCR products were sequenced using an ABI 3730 sequencer (Applied Biosystems, Foster City, CA); and the data obtained were compared with the reported sequences retrieved from GenBank. A strain with positive string test, positive K1 positive K1 capsular polysaccharide gene and one or more of the virulence was defined as hvKP.

**Table 1:** **List of primers for detection of *Klebsiella pneumoniae* virulence genes**.

**Gene**	**Primer**	**Primer sequence**	**Amplicon (bp)**	**Annealing temperature (°C)**	**References**
K1	K1-F	GTAGGTATTGCAAGCCATGC	1047	55	Fang et al., 2007
	K1-R	GCCCAGGTTAATGAATCCGT			
K2	K2-F	GACCCGATATTCATACTTGACAGAG	641	57	Turton et al., 2008
	K2-R	CCTGAAGTAAAATCGTAAATAGATGGC			
K5	K5-F	TGGTAGTGATGCTCGCGA	280	55	Turton et al., 2008
	K5-R	CCTGAACCCACCCCAATC			
K20	K20-F	CGGTGCTACAGTGCATCATT	741	55	Fang et al., 2007
	K20-R	GTTATACGATGCTCAGTCGC			
K54	K54-F	CATTAGCTCAGTGGTTGGCT	881	55	Fang et al., 2007
	K54-R	GCTTGACAAACACCATAGCAG			
K57	K57-F	CTCAGGGCTAGAAGTGTCAT	1037	55	Fang et al., 2007
	K57-R	CACTAACCCAGAAAGTCGAG			
*wcaG*	*wcaG*-F	GGTTGGKTCAGCAATCGTA	169	53	Turton et al., 2010
	*wcaG*-R	ACTATTCCGCCAACTTTTGC			
*rmpA*	*rmpA*-F	ACTGGGCTACCTCTGCTTCA	535	50	Nadasy et al., 2007
	*rmpA*-R	CTTGCATGAGCCATCTTTCA			
*magA*	*magA*-F	GGTGCTCTTTACATCATTGC	1282	53	Fang et al., 2004
	*magA*-R	GCAATGGCCATTTGCGTTAG			
Aerobactin	Aerobactin-F	GCATAGGCGGATACGAACAT	556	55	Yu et al., 2008
	Aerobactin-R	CACAGGGCAATTGCTTACCT			

### Molecular typing

Epidemiological relatedness of the K1 strains was studied by pulsed-field gel electrophoresis (PFGE) and multilocus sequence typing (MLST). Twenty-three K1-positive *K. pneumoniae* strains were genotyped by PFGE following the PulseNet protocol provided by the website of the U.S. Centers for Disease Control and Prevention (http://www.cdc.gov/pulsenet/pathogens/index.html). The bacterial cells were digested with the XbaI restriction enzyme and were separated in a Rotaphor System 6.0 instrument (Whatman Biometra). Banding patterns of 23 isolates were analyzed using the UVIBand software program (UVItec Ltd., Cambridge, United Kingdom), and the degree of sequence homology was calculated (unweighted pair group method using average linkages [UPGMA],0.5% Master Lane). MLST was performed on representative isolates of each clonal type. Seven housekeeping genes of *K. pneumoniae* (*gapA, infB, mdh, pgi, phoE, rpoB*, and *tonB*) were amplified, sequenced and analyzed (Turton et al., 2007). Allele sequences and sequence types (STs) were analyzed according to the MLST database (http://pubmlst.org/).

### Protein extraction

*K.pneumoniae* isolates were inoculated onto Columbia blood agar (Oxoid, Cambridge, UK) containing 5% sheep blood and incubated 18–24 h at 35°C. Several uniform colonies from fresh plates were re-suspended into 300 μl of distilled water. After addition of 900 μl ethanol, the extraction tube was centrifuged at 12,000 × g for 2 min, the supernatant was then discarded. The bacterial pellet was re-suspended in 50 μl 70% formic acid. Before centrifuging again, 50 μl acetonitrile was added. After 2 min centrifugation at 12,000 × g, 1 μl of supernatant was spotted onto the ground steel target and dried at room temperature. One microliter of alpha-cyano-4-hydroxycinnamic acid (CHCA) was overlaid and dried again.

### MALDI-TOF MS analysis

MALDI-TOF MS analysis was performed on a Bruker MicroFlex LT mass spectrometer (Bruker Daltonics). Spectra were acquired according to the manufacturer's recommendations, mass range was from 2000 to 20,000 Da and the laser intensity was kept constant. Mass spectra were analyzed by the Biotyper 3.0 software and library (version 3.0, Bruker Daltonics). Identification score criteria used followed those recommended by the manufacturer: a score of >2.000 indicated species-level identification, a score of 1.700–1.999 indicated identification to the genus level, and a score of < 1.700 was interpreted as inconclusive.

### Data analysis

The ClinProTools software (v3.0; Bruker Daltonics) was used for peak analysis. Models were generated using all four available algorithms, genetic algorithm (GA), support vector machine (SVM), supervised neural network (SNN), and quick classifier (QC), followed by comparison to each other. For each model, the recognition capability and cross validation were calculated to demonstrate the sensitivity and specificity of the model, statistical analysis were obtained by the most reliable algorithm model. The receiver operating characteristic (ROC) curves for each of the peaks of interest were obtained from the ClinProTools software. The area under curve (AUC) was used to evaluate the performance of each algorithm.

## Results

### Bacterial isolates used in this study

Forty K1hvKP strains were identified from 438 clinical non-repeated *K. pneumoniae* strains isolated from patients in Zhejiang Provincial hospital. Twenty-three out of the 40 K1 hvKP and 20 non-K1 isolates were randomly chosen for the development of MALDI-TOF MS method. The rest of 17 K1 isolates along with another 20 non-K1 isolates were used to evaluate the differentiation power of the MALDI-TOF MS method.

### Molecular features of K1 and non-K1 strains

**Figure 1:**
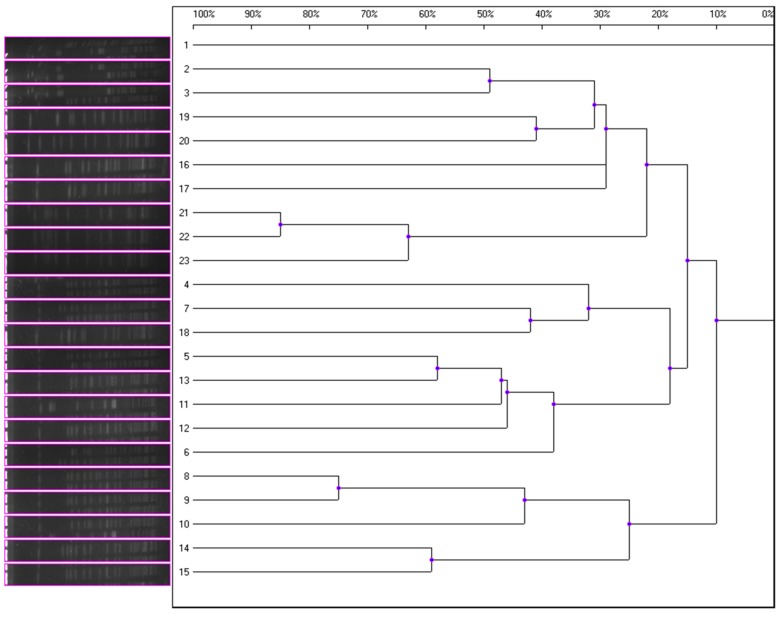
**Magnified dendrogram (representation of hierarchical cluster analysis) of 23 K1hvKP isolates**.

All 40 K1 hvKP contained K1 capsular gene and at least *magA* and Aerobactin genes. MLST analysis identified four STs including ST23, ST520, ST700, and ST1552 with ST23 the most dominant one (20 ST23 out of a total of 23K1 hvKP). In addition to *magA* and Aerobactin genes, all ST23 K1 hvKP also contained *wcaG* and *rmpA*; one ST700 contained *wcaG* (Table 2). Within the 20 ST23 K1 hvKP strains, only two (21 and 23) were shown to be identical by PFGE analysis (Figure 1). In contrast, the non-K1 strains are found to exhibit a wide range of genetic diversity with 14 different STs being identified in the 20 non-K1 *K. pneumoniae*. None of the non-K1 *K. pneumoniae* harbored any of the four genetic markers suggesting they are not hvKP strains (Table 2). Notably, STs in K1 hvKP and non-K1 *K. pneumoniae* were different suggesting the close association of STs to K1 hvKP in particular ST23.

**Table 2:** **Prevalence of ST types and known virulence genes in K1 hvKP and non-K1 *K. pneumoniae* strains**.

**Capsule type**	**ST types**	**No. of isolates**	**Other virulence genes**			
			***wcaG***	***rmpA***	***magA***	**Aerobactin**
K1	23	20	+	+	+	+
	520	1	−	−	+	+
	700	1	+	−	+	+
	1552	1	−	−	+	+
non-K1	12	1	−	−	−	−
	34	1	−	−	−	−
	35	4	−	−	−	−
	36	1	−	−	−	−
	37	2	−	−	−	−
	138	2	−	−	−	−
	705	1	−	−	−	−
	753	1	−	−	−	−
	875	1	−	−	−	−
	983	1	−	−	−	−
	1411	1	−	−	−	−
	1547	2	−	−	−	−
	1548	1	−	−	−	−
	1551	1	−	−	−	−

### Data analysis by MALDI-TOF MS

Of the 43 *K. pneumoniae* modeling isolates analyzed, all (100%) were identified correctly as *K. pneumoniae* by MALDI-TOF MS. The models generated using four standard algorithms, GA, SVM, SNN, and QC, for comparing K1 with non-K1 isolates, were shown in Table 3. The use of the QC standard algorithm resulted in the lowest scores, whereas GA standard algorithm exhibited the highest specificity (recognition capability at 100%) when compared to the SVM standard algorithm (recognition capability at 97.83%). Yet SVM gave the highest sensitivity at 83.45% among four models, which is higher than the rate of 73.87% for GA. However, the specificity of SVM is slightly lower than GA being at 97.83%. Overall, SVM and SNN were the most reliable model for differentiating between K1 from classic or non-K1 isolates. The study showed that peaks or integration regions chosen for differentiation of K1 *K. pneumoniae* status by all four of the models were similar. The important peaks identified by the SVM model for K1 were 14, 31, 33, and 34. The peak statistics for these peaks are shown in Table 4.

**Table 3:** **Specificity and sensitivity of different algorithms models for differentiation between K1 and non-K1 *K. pneumoniae* isolates**.

**Algorithms model**	**Assay development**		**Assay validation**			
	**Cross validation**	**Recognition capability**	**Specificity (true negative)**	**95% CI of Specificity**	**Sensitivity (true positive)**	**95% CI of Sensitivity**
GA	100%	73.9%	16/17 (94.1%)	86.5–100%	17/20 (85.0%)	73.5–96.5%
SVM	97.8%	83.5%	16/17 (94.1%)	86.5–100%	18/20 (90.0%)	80.3–99.7%
SNN	100%	81.4%	16/17 (94.1%)	86.5–100%	18/20 (90.0%)	80.3–99.7%
QC	85.7%	70.7%	15/17 (88.3%)	77.9–98.6%	16/20 (80.0%)	67.1–92.9%

**Table 4:** ClinProTools peak statistics for the four peaks of interest in both K1 hvKP and non-K1 K. pneumoniae^*^.

**Peak**	**Mass**	**DAve**	**PTTA**	**PWKW**	**PAD**	**Ave1**	**Ave2**	**SD1**	**SD2**	**CV1**	**CV2**
14	3586.58	2.1	0.000339	0.000258	0.0729	3.1	5.2	1.23	1.48	39.71	28.36
31	4744.66	8.27	0.000164	0.000258	0.0843	12.91	21.18	5.41	4.61	41.94	21.74
33	5044.84	1.62	0.00036	0.000819	0.225	3.07	4.69	1.34	0.83	43.6	17.77
34	5148.93	2.59	0.000198	0.000525	0.0729	3.53	6.12	1.51	1.68	42.72	27.49

* *Sort mode, delta average arithmetic; peak, peak index; mass, m/z value; DAve, difference between the maximal and the minimal average peak area/intensity of all classes; PTTA, P-value of t-test; PWKW, P-value of Wilcoxon (preferable for abnormally distributed data); PAD, P-value of Anderson-Darling test (range, 0–1; 0, abnormally distributed; 1, normally distributed); Avg1 and Avg2, peak area/intensity average of class 1 (K1 K. pneumoniae isolates) and class 2 (non-K1 K. pneumoniae isolates), respectively; SD1 and SD2, standard deviations of the peak area/intensity average of class 1 and class 2, respectively; CV1 and CV2, coefficient of variation (in percentage) of class 1 and class 2, respectively*.

The low *P*-values for the Anderson-Darling test (PAD) are evidence of the abnormal distribution of the data obtained. Therefore, the *P*-value of the PWKW (*P*-value from combined Wilcoxon rank-sum test and Kruskal-Wallis test) is preferred over the PTTA (*p*-value of *t*-test) (as this is preferable for normally distributed data). The low *P*-values obtained from PWKW (all were < 0.05) indicated that the observed intensity differences of the individual peaks are highly statistically significant (i.e., the lower the *P*-value is, the higher the chance that a respective peak signal is suited to differentiate between the two classes) (Table 4).

**Figure 2:**
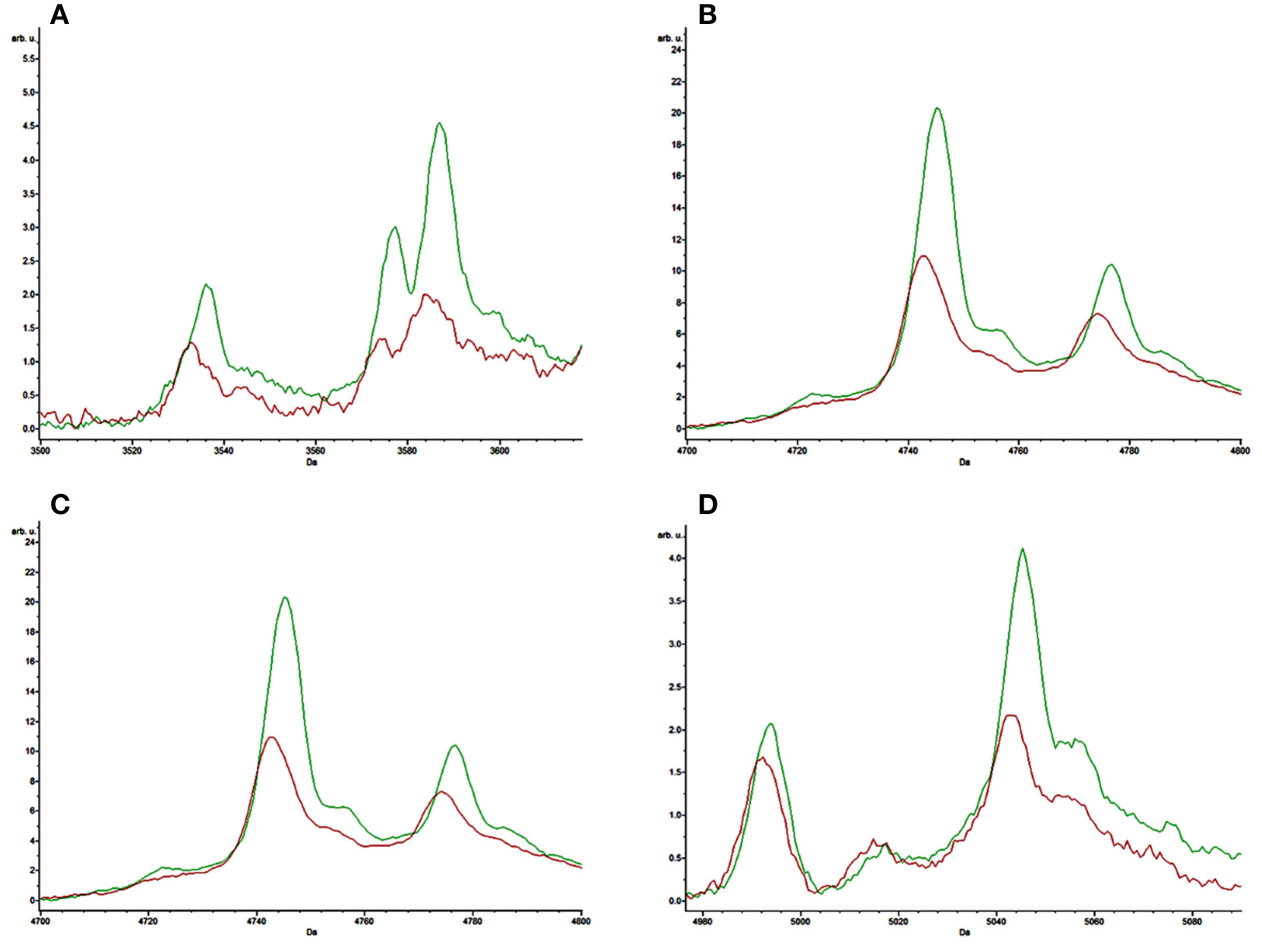
**Representative comparison of the average spectra of the K1 hvKP isolates (red) and non-K1 *K. pneumoniae* isolates (green)**. **(A–D)** are the representative spectra of K1 hvKP and non-K1 *K. pneumoniae*.

The receiver operating characteristic (ROC) curves for each of the peaks of interest generated by the SVM model were also obtained from the ClinProTools software. The area under curve (AUC) can reflect the confidence level of each peak in identifying the sensitivity and specificity of virulent strains and non-K1 strains group, with an AUC of 0.5 representing purely random chance and an AUC of 1 indicating a perfect test (100% sensitivity and specificity; Table 4). All characteristic peaks used for distinguishing virulent from non-K1 strains were >0.8, confirming that these peaks can be used to differentiate K1 from non-K1 *K. pneumoniae* isolates with high accuracy. To demonstrate the differences visually, four representative peaks are shown in Figure 2, in which the spectra of the K1 and non-K1 *K. pneumoniae* isolates were distinguishable.

### Prospective validation

Another 20 randomly selected non-K1 *K. pneumoniae* and 17 remaining K1-positive hvKP isolates were chosen to perform the prospective verification. The verification result indicated that the accuracy of K1 and non-K1 identification was 94.1 and 90.0%, respectively, according to the SVM model suggesting the high accuracy of MALDI-TOF MS method to rapid identification of K1 hvKP (Table 3).

## Discussion

Hospital-acquired *K. pneumoniae* clinical isolates normally exhibit relatively low-level virulence, whereas in most cases of community-acquired pneumonia, *Klebsiella* isolates are generally highly virulent and known to produce mucoid colonies. Recent studies indicated that hvKP induced liver abscess was a new type of invasive infections (Siu et al., 2012), and that hvKP can not only cause liver abscess, but also metastatic infections such as bacteremia, meningitis, endophthalmitis, and necrosis fasciitis, often resulting in fatality in severe cases. A number of virulence factors have been identified in pathogenic *K. pneumoniae*, including capsular polysaccharide, mucus related gene A (mucoviscosity-associated gene A, *magA*), mucous phenotype A regulator gene (regulator of mucoid phenotype gene A, *rmpA*), and, aerobactin. Capsular polysaccharide is considered one of the major contributive factors of virulence in *K. pneumoniae*, promoting biofilm formation and exhibiting anti-opsonin effect which can help combat the host immune response when expressed in the host body (Cortés et al., 2002). To date, *K. pneumoniae* producing K1 type capsule is the major pathogen causing community-acquired lung infections in Asia (Lin et al., 2015).

Current hvKP detection methods include PCR amplification, multilocus sequence typing (MLST), pulsed field gel electrophoresis (PFGE), and the proteomics approach, which are complicated and time consuming, and demand technical competency. Since hvKP is highly virulent, causing high mobility and mortality among infected patients, a rapid and accurate clinical identification method is urgently needed to guide proper treatment of patients infected by hvKP. MALDI-TOF MS is a revolutionary technique for clinical bacteria identification. It has high power to identify clinical common bacteria, yeasts and fungi and has been shown to be used to identify antimicrobial resistance and virulence gene products. Based on the specific peaks, MALDI TOF mass spectrometry can be used to identify *bla*_KPC_-positive *K. pneumoniae*, van B positive *Enterococci feces* and virulence factors of *S. aureus* such as delta-toxin and PSM-mec (Josten et al., 2014).

In this study, we used 20 non-K1 *K. pneumoniae* isolates with various ST patterns as control strains. Despite exhibiting different MS spectra, however, we were still able to differentiate between the non-K1 *K. pneumoniae* and K1 hvKP strains, producing results which were highly consistent with data of MLST tests and genotyping of known virulence genes. Although no specific spectrum has been identified for K1 hvKP, four peaks at *m/z* of 3587, 4745, 5045, and 5149 were shown to be significantly (*P* < 0.05) stronger among the non-K1 strains when compared to the K1 hvKP strains. In addition, ROC of AUC reached >0.8, suggesting that these parameters have high predictive value for distinguishing between K1 and non-K1 *K. pneumoniae*. Since these peaks could also be reproduced in other clinical validation isolates with similar accuracy and sensitivity, we conclude that MALDI-TOF MS based method is a simple approach for rapidly and accurately identifying K1 *K. pneumoniae*, which have become prevalent and clinically significant, thereby greatly facilitating prompt and efficient treatment of infections caused by this notorious pathogen. The limitation of current study includes the 94.1 and 90.0% discrimination power for K1 hvKP and non-K1 KP, respectively, which may result in some false positive results, and relatively small number of isolates for assay development and validation, increasing of which may significantly improve the accuracy and specificity of the method. The study at this stage is only a preliminary investigation demonstrating the possibility to detect K1 hvKP by MALDI-TOF MS and requires extensive clinical validation before it can be used as a validated clinical method.

### Conflict of interest statement

The authors declare that the research was conducted in the absence of any commercial or financial relationships that could be construed as a potential conflict of interest.
